# UV/H_2_O_2_-Degraded Polysaccharides from *Sargassum fusiforme*: Purification, Structural Properties, and Anti-Inflammatory Activity

**DOI:** 10.3390/md21110561

**Published:** 2023-10-26

**Authors:** Shiyuan Chang, Xiaoyong Chen, Yifan Chen, Lijun You, Kseniya Hileuskaya

**Affiliations:** 1 School of Food Science and Engineering, South China University of Technology, Guangzhou 510640, China; amsychang@163.com (S.C.); chenxiaoyong522@163.com (X.C.); yifaaan@163.com (Y.C.); 2 College of Food Science, Southwest University, Chongqing 400715, China; 3 Overseas Expertise Introduction Center for Food Nutrition and Human Health (111 Center), Guangzhou 510640, China; 4 Institute of Chemistry of New Materials, National Academy of Sciences of Belarus, 36F. Skaryna Str., 220141 Minsk, Belarus; k_hilevskay@mail.ru

**Keywords:** polysaccharides, *Sargassum fusiforme*, degradation, UV/H_2_O_2_, anti-inflammatory activity

## Abstract

The main purpose of this study was to analyze the structural properties and anti-inflammatory activity of the purified fractions derived from UV/H_2_O_2_-degraded polysaccharides from *Sargassum fusiforme*. Results indicated that two fractions with different monosaccharide compositions and morphological characteristics, PT-0.25 (yield 39.5%) and PT-0.5 (yield 23.9%), were obtained. The average molecular weights of PT-0.25 and PT-0.5 were 14.52 kDa and 22.89 kDa, respectively. In addition, PT-0.5 exhibited better anti-inflammatory activity with a clear dose dependence. The mechanism was associated with the inhibition of LPS-activated Toll-like receptor 4-mediated inflammatory pathways in RAW 264.7 cells. The results showed that PT-0.5 was a complex polysaccharide mainly composed of 4-Fuc*p*, t-Man*p*, 6-Gal*p*, t-Fuc*p*, and 3,4-GlcA*p*. These results would provide theoretical support for studying the structural properties and biological activities of UV/H_2_O_2_-degraded polysaccharides.

## 1. Introduction

Many polysaccharides from seaweed have attracted a lot of attention in industries, including foods, cosmetics, and medicine, since they exhibited a great variety of bioactivities [1]. However, their weak solubility and bioavailability limited the application, so various modification methods have been used to change their physicochemical and structural properties [2]. Current studies have shown that the degradation methods of acid, high-temperature, enzyme, or free radical could effectively decrease the molecular weight (M*_W_*) of polysaccharides [2]. Moreover, degradation of polysaccharides usually has a significant effect on biological activity [3,4,5]. For example, ascorbic acid combined with H_2_O_2_-degraded polysaccharides from *Lycium barbarum* L. leaves increased the gastrointestinal transport and bioaccessibility of endogenous minerals [6]. Polysaccharides from *Laminaria japonica* were prepared using thermo-acid pretreatment and alginate lyase hydrolysis, which can ameliorate intestinal damage and have hypolipidemic effects [7]. It has been demonstrated that changes in a polysaccharide’s sulfate group, monosaccharide composition, M_W_, branching degree, glycosidic–linkage composition, and conformation correlate with the changes in its bioactivity [8]. Therefore, purification and identification of degraded polysaccharides help explore compound databases with desirable bioactivities.

*Sargassum fusiforme*, a species of brown seaweed, provides both dietary benefits and practical medicinal value. Some studies have indicated that degradation had a significant impact on enhancing the health benefits of polysaccharides from *Sargassum fusiforme*. For example, ascorbic acid combined with H_2_O_2_ treatment improved its antioxidant activity [9]. The pectinase and glucoamylase treatment improved its immunoregulatory activity [10]. Our earlier research demonstrated that PSF can be efficiently degraded via UV/H_2_O_2_ (UV combined with H_2_O_2_) treatment [11], and PSF-T2, a UV/H_2_O_2_-degraded polysaccharide from *Sargassum fusiforme*, had good anti-inflammatory activity [12,13]. However, little structural information on PSF after UV/H_2_O_2_ treatment was known. Therefore, in this study, PSF-T2 was first isolated, and then the obtained purified fractions were analyzed for their structural properties and anti-inflammatory activity.

## 2. Results and Discussion

### 2.1. Isolation and Purification of Polysaccharides and Their Molecular Weight

**Figure 1:**
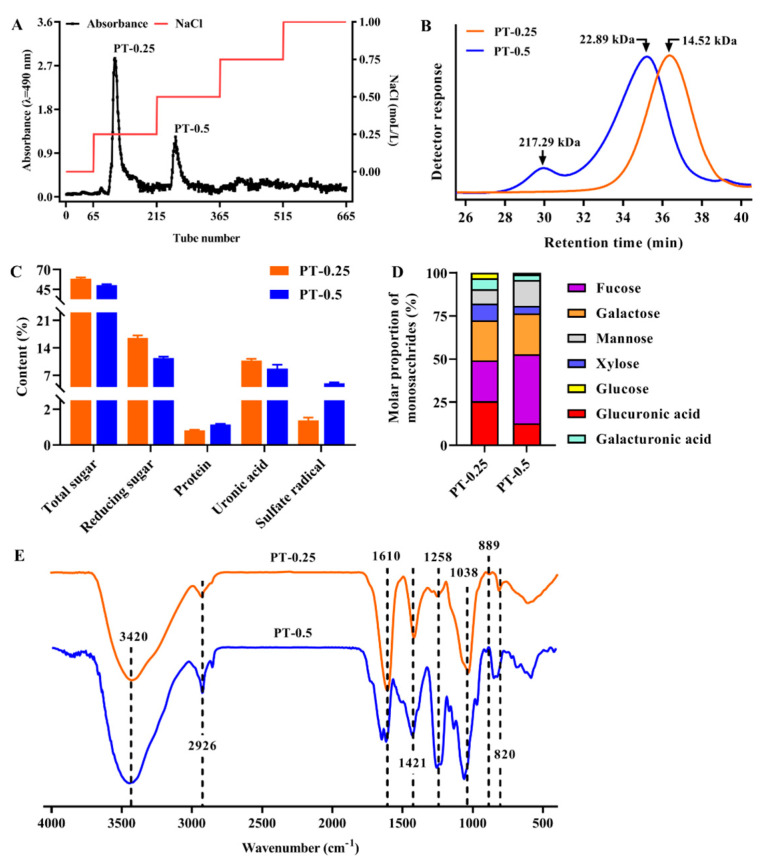
Elution curve of PSF-T2 using DEAE-650M (**A**); the M_W_ (**B**); chemical composition (**C**); monosaccharide composition (**D**); and FT-IR spectra (**E**) of purified fractions.

As shown in Figure 1A, two fractions, named PT-0.25 and PT-0.5, were obtained via a Toyopearl-DEAE 650 M column separation (Tosoh, Tokyo, Japan). PT-0.25 and PT-0.5 accounted for a yield of about 39.50% and 23.90% of PSF-T2, respectively. The relationship between retention time and molecular weight was calculated as Log M_W_ = a + b*x* + c*x*^2^ (a = 12.32, b = −0.2739, c = 1.354 × 10^−3^, R^2^ = 0.9999), where *x* represents the retention time (min). As shown in Figure 1B, the M_W_ of PT-0.25 was smaller than that of PT-0.5. Specifically, the M_W_ of PT-0.25 had a peak between the retention times of 28 and 38 min, and its M_W_ was 14.52 kDa. The M_W_ of PT-0.5 had two peaks between the retention times of 28 and 38 min, where the M_W_ of the large peak was 22.89 kDa, and the M_W_ of the small peak was 217.29 kDa.

### 2.2. Chemical Composition and Monosaccharide Composition of Purified Fractions

As shown in Figure 1C, compared with the chemical compositions of PT-0.5, PT-0.25 had higher total sugar (58.56 ± 1.60%), reducing sugar (16.54 ± 0.63%), and uronic acid (10.78 ± 0.44%), as well as lower protein (0.82 ± 0.04%) and sulfate radical (1.38 ± 0.16%) contents. Correspondingly, PT-0.5 was 50.33 ± 1.40%, 1.15 ± 0.03%, 11.39 ± 0.38%, 8.70 ± 1.02%, and 5.00 ± 0.23%, respectively.

The monosaccharide composition analysis showed that PT-0.25 and PT-0.5 were composed of seven different kinds of monosaccharides, fucose, glucuronic acid, and galactose were their main constituents, accounting for about 75%, while xylose, mannose, galacturonic acid, and glucose were relatively rare (Figure 1D). In addition, the content of fucose in PT-0.5 was higher than that in PT-0.25, and the main components of the above two fractions were both acidic heteropolysaccharides.

### 2.3. FT-IR Spectra of Purified Fractions

As shown in Figure 1E, the peaks at 2926 cm^−1^ and 3420 cm^−1^ were caused by the stretching vibrations of C-H and O-H, respectively [14]. The peaks at 1421 cm^−1^ and 1610 cm^−1^ were caused by C-H bending and the C=O stretching vibration, respectively [15]. The peak at 1038 cm^−1^ was caused by C-O-C, indicating the presence of a pyranose ring, which could be derived from fucoidan units [16]. Moreover, no absorption peak was observed at 788 and 802 cm^−1^, which was attributed to the guluronic acid and mannuronic acid, respectively. The peak at 1258 cm^−1^ was assigned to the stretching vibrations of S=O, indicating the presence of the sulfate group [17]. The absorption peak at 1280 nm of PT-0.25 was weaker than PT-0.5, which was consistent with the content of the sulfate radical measured above. The absorption at 820 cm^−1^ and 889 cm^−1^ suggested that PT-0.25 and PT-0.5 had β-configuration and α-configuration [18]. These results indicated that PT-0.25 and PT-0.5 were both sulfated polysaccharides, which had α- and β-type glycosidic linkages.

### 2.4. Morphological Characterization of Purified Fractions

**Figure 2:**
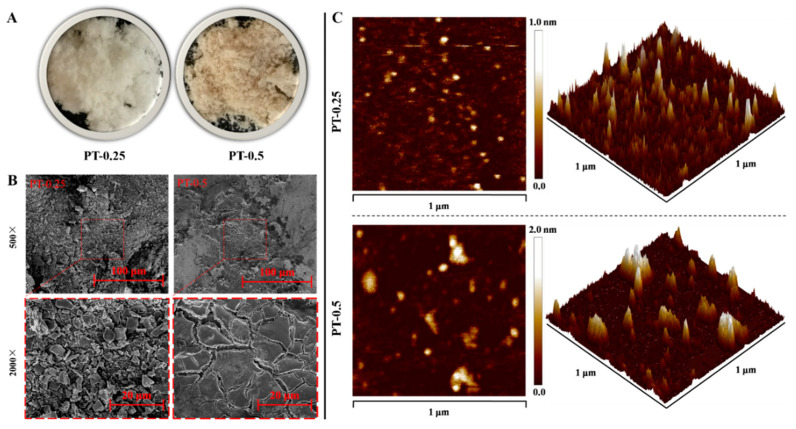
Photograph images (**A**), SEM images (**B**), and AFM (**C**) images of purified fractions.

As shown in Figure 2A, lyophilized PT-0.25 and PT-0.5 were white and yellowish, respectively. SEM showed that the surface morphologies of PT-0.25 at magnifications of 500×, and 2000× presented as relatively loose, non-porous, and irregularly flaky with a non-uniform size, but the morphology of PT-0.5 was characterized by an irregular lamellar structure with a smooth and non-porous surface (Figure 2B). The result differed from that of a previous study in which a polysaccharide from *Sargassum fusiforme*, obtained from ultrasound-assisted enzymatic extraction, had a rough surface with small holes [19]. These differences indicated that the morphological characterization of polysaccharides was influenced by extraction, purification, and degradation, which was consistent with the previous study [20].

AFM is one of the useful tools to characterize the three-dimensional structure and nanoscale microstructure of polysaccharides [21]. As shown in Figure 2C, the planar images of PT-0.25 and PT-0.5 presented circles with inhomogeneous size and shape. Among them, the circular homogeneity of PT-0.5 was worse than that of PT-0.25. From three-dimensional images, it can be seen that PT-0.25 and PT-0.5 had a flame-like distribution. The height of PT-0.5 was higher than that of PT-0.25, indicating that it was more rough. In addition, the concentrations of PT-0.25 and PT-0.5 used for AFM observation in this study were low, indicating that the overlap and entanglement between polysaccharide molecules were not the main factors causing their spatial conformational differences. Therefore, the aggregates of PT-0.5 are also larger than those of PT-0.25, indicating that the intermolecular interactions are greater.

### 2.5. Cell Viability of Purified Fractions

**Figure 3:**
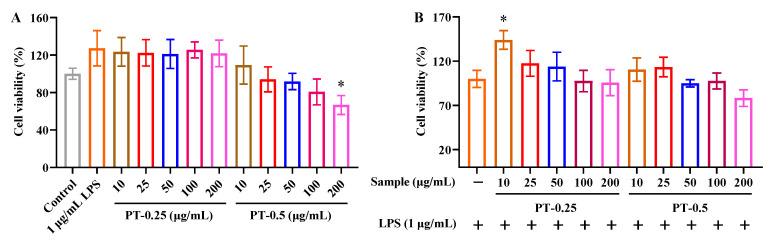
Cell viability of purified fractions in RAW 264.7 cells (**A**) and LPS-stimulated RAW 264.7 cells (**B**); * *p* < 0.05 compared with control group or LPS alone treatment. The plus and minus mean addition or not.

As shown in Figure 3A, LPS and the purified fractions’ treatments for 24 h were non-toxic in RAW 264.7 cells except for the 200 μg/mL PT-0.5 treatment. In addition, the cytotoxicity of PT-0.25 was lower than that of PT-0.5 at the same concentration.

As shown in Figure 3B, when RAW 264.7 cells were sequentially treated with the purified fractions and LPS for 6 h and 24 h, respectively, the purified fractions had no significant effects on the viability of RAW 264.7 cells except for 10 μg/mL PT-0.25. Considering the relatively low cell viability of the 200 μg/mL PT-0.5 treatment for LPS-stimulated RAW 264.7 cells, these concentrations (25, 50, and 100 μg/mL) of purified fractions were selected for the following study.

### 2.6. Purified Fractions Inhibited the Secretion of Pro-Inflammatory Mediators

**Figure 4:**
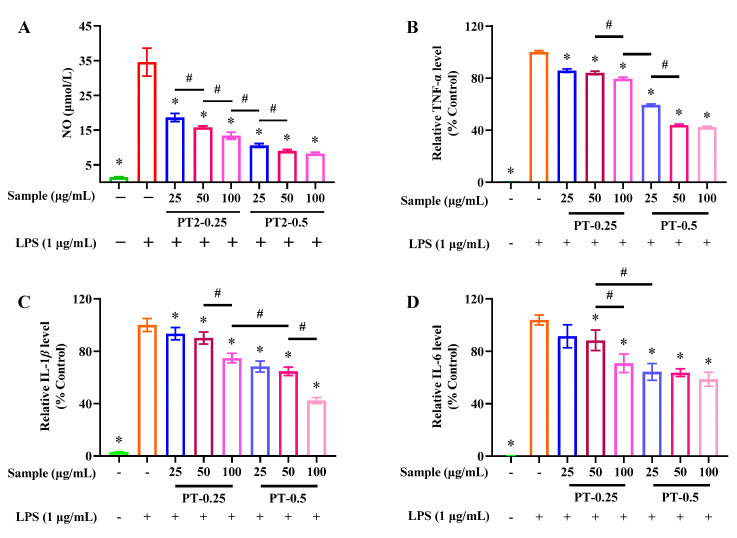
Levels of NO (**A**), TNF-α (**B**), IL-1β (**C**) and IL-6 (**D**) after purified fractions treatment in LPS-stimulated RAW 264.7 cells; * *p* < 0.05 and ^#^ *p* < 0.05 compared with LPS alone treatment. The plus and minus mean addition or not.

As shown in Figure 4, LPS treatment alone for 24 h significantly promoted the secretion of NO, IL-1β, IL-6, and TNF-α in RAW 264.7 cells, which was mainly attributed to the ability of LPS to bind to the TLR 4 receptor and induce inflammatory responses. However, the secretion of the above inflammatory mediators was significantly inhibited after 6 h of 25–100 μg/mL PT-0.25 or PT-0.5 pretreatment except for 25 μg/mL PT-0.25 on IL-6 secretion, and there is a dose dependence of their inhibitory effects. In addition, PT-0.5 could better inhibit the secretion of the above-mentioned pro-inflammatory mediators than PT-0.25 at the same dose, indicating that PT-0.5 had better anti-inflammatory activity. Fucoidan isolated from *Sargassum autumnale* have been reported to possess strong decreased pro-inflammatory cytokines’ IL-1β, TNF-α, and IL-6 expression in LPS-induced cells [22]. In addition, a fucoidan with a molecular weight of 102.67 kDa isolated from an enzymatic digest of *Sargassum fusiforme* was also proven to inhibit the production of TNF-α, NO, IL-1β, and IL-6 [23].

### 2.7. Purified Fractions Inhibited the Expression of Related Pro-Inflammatory Genes

**Figure 5:**
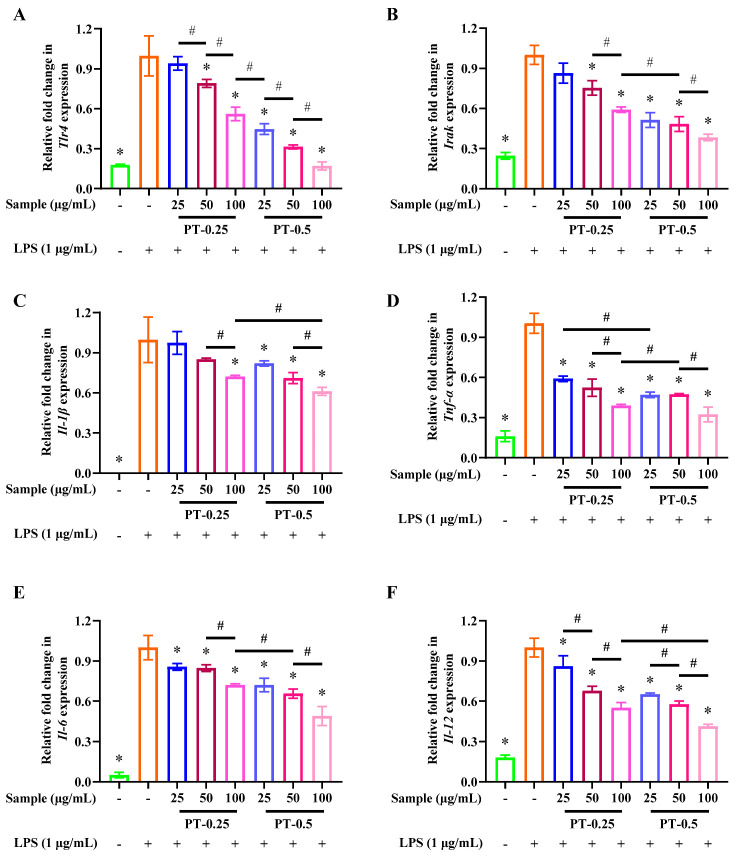
Levels of *Tlr4* (**A**), *Irak* (**B**), *Il-1β* (**C**), *Tnf-α* (**D**), *Il-6* (**E**) and *Il-12* (**F**) expression after treatment with the two purified fractions in LPS-stimulated RAW 264.7 cells; * *p* < 0.05, # *p* < 0.05 compared with LPS alone treatment. The plus and minus mean addition or not.

Based on the above results, the anti-inflammatory activities of PT-0.25 and PT-0.5 were further validated by analyzing inflammation-related gene expression. As shown in Figure 5, LPS treatment alone for 24 h significantly up-regulated the expression of *Tlr4*, *Irak*, *Tnf-α*, *Il-1β*, *Il-6*, and *Il-12* in RAW 264.7 cells, where their expression levels can be inhibited by PT-0.25 and PT-0.5, and there is a dose dependence of their inhibitory effects on the expression of related pro-inflammatory genes. Jayasinghe et al. isolated a fucoidan with low molecular weight (8177 Da) from the brown alga *Sargassum siliquastrum*, of which it could also downregulate the mRNA expression of IL-1β, IL-6, and TNF-α [24]. In addition, compared with the equal dose of the PT-0.25 treatment, PT-0.5 can better inhibit the expression of the above-mentioned genes. Therefore, these results further showed that PT-0.5 had better anti-inflammatory activity and that its anti-inflammatory mechanism was related to the inhibition of the LPS-TLR4 signaling pathway. 

### 2.8. Methylation Analysis of PT-0.5 Fraction

Considering that PT-0.5 had better anti-inflammatory activity, we further analyzed its glycosidic linkage. As shown in Table 1, PT-0.5 contained three non-reducing sugars, corresponding to the terminal sugar of the branch (t-Fuc*p*, t-Man*p*, and t-GalA*p*), where six sugars were part of the linear chain (4-Xyl*p*, 2-Gal*p*, 4-Man*p*, 6-Gal*p*, 4-Gal*p*, and 4-Fuc*p*) and the point of branching was glucuronic acid (3,4-GlcA*p*). These results showed that PT-0.5 was a complex-branched polysaccharide. In addition, the fucose residues were mainly in the main chain of PT-0.5, and the backbone of PT-0.5 mainly contained 4-Fuc*p*, t-Man*p*, t-Fuc*p*, 6-Gal*p*, and 3,4-GlcA*p*. The types of sugar residues were in general agreement with those of the monosaccharide composition analysis.

### 2.9. NMR Spectra of Purified Fraction with Best Anti-Inflammatory Activity

To further obtain more structural information, we initially proposed studying PT-0.5 using the 1D and 2D NMR spectra. However, due to the weak signals in the ^13^C NMR spectra, the 2D NMR for the in-depth analysis was not performed.

**Figure 6:**
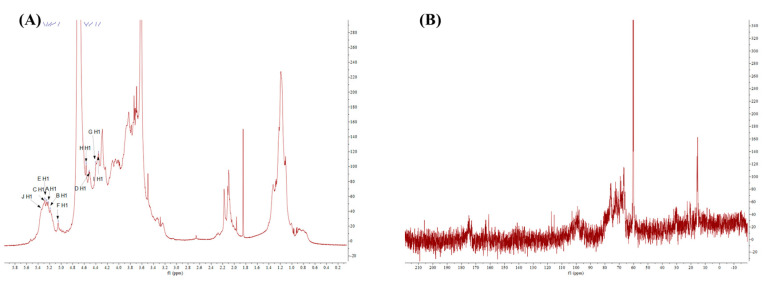
^1^D NMR spectra of PT-0.5: ^1^H NMR (**A**), ^13^C NMR (**B**).

In the ^1^H NMR spectra (Figure 6A), multiple signals were presented in the regions of anomeric protons (4.4–5.5 ppm) [25], such as these signals at δ 5.35, δ 5.25, δ 5.19, δ 5.05, δ 4.57, and δ 4.52 ppm. These signals indicated that PT-0.5 had *α*-configuration and β-configuration (δ > 5.00 ppm and δ < 5.00 ppm indicated the presence of α-configuration and β-configuration, respectively), which was in agreement with the results of FT-IR. In the high field (1.1–4.3 ppm), the signal around δ 1.17 ppm likely originated from the proton of methyl in fucose residues [26]. The signal at δ 2.09 ppm belonged to the proton of methyl in the acetyl group or/and the *N*-acetyl group [27,28]. In addition, the signals at 4.10 and 4.00 ppm corresponded to methylene hydrogen in carboxymethyl [29]. Compared with the reported studies, we have indicated the chemical shifts in the spectrum of ^1^H.

In the ^13^C NMR spectra (Figure 6B), most signals were not obvious, especially in the regions of anomeric carbon (90–110 ppm), so the ^13^C NMR spectra could not give enough carbon information for further analysis. So, we increased the polysaccharide concentration while maintaining mobility to enhance the signal strength, but the results did not improve significantly. From the ^13^C NMR spectra, only two signals were strong, as their chemical shifts were respectively at δ 15.42  ppm and δ 60.25  ppm. The signals at 15.42  ppm indicated the existence of fucose [30]. The signals at 60.25  ppm corresponded to C-6 in the monosaccharide residues.

## 3. Materials and Methods

### 3.1. Preparation and Purification of PSF-T2

PSF-T2 was prepared using a pre-established method [12]. Briefly, the *Sargassum fusiforme* was collected from the Dongtou District, Wenzhou, Zhejiang, China. Polysaccharides of *Sargassum fusiforme* (PSF) were prepared via hot water extraction (1:50, m:v) and 80% ethanol precipitation, followed by sufficient ethanol removal and lyophilization. PSF was further processed by UV irradiation at 313 nm (HOPE-MED 814, Tianjin Hepu Company, Tianjin, China) combined with H_2_O_2_ treatment for 2 h at 25 °C to obtained PSF-T2, PSF and H_2_O_2_ concentrations were 2.5 mg/mL and 100 mmol/L, respectively. Then, 20 mL of 10 mg/mL PSF-T2 was loaded on a chromatographic column (26 mm × 600 mm i.d.) containing 200 mL Toyopearl-DEAE 650 M (Tosoh, Tokyo, Japan). Stepwise gradient elution with 0, 0.25, 0.5, 0.75, and 1.0 mol/L NaCl in 50 mmol/L Tris-HCl was performed and the flow rate was 1 mL/min. The eluent was collected in tubes (5 mL/tube) and its total sugar was determined. Finally, the collected target gradient eluate was concentrated, dialyzed, centrifuged, freeze-dried, and kept at cryogenic temperatures until use.

### 3.2. Chemical Composition and Molecular Weight Analysis

Total sugar was detected by using the phenol–sulfuric acid method [31]. Protein was detected by using the Bradford method [32]. Uronic acid was detected by using the carbazole method [33]. Reducing sugar was detected by using the 3,5-dinitrosalicylic acid method [34]. Sulfate was detected by using the BaCl_2_–gelatin method [35]. The reference standards for the above tests were fucose, bovine serum albumin, glucuronic acid, glucose, and potassium sulfate, respectively. The M_W_ was detected by using high-performance gel-permeation chromatography, and dextrans with different molecular weights were used as the standards [11]. Briefly, polysaccharide samples were prepared as a 2 mg/mL solution and filtered through a 0.22 μm millipore filter. An aliquot of 20 μL of the sample solution was injected into a TSK-GEL G-6000 PWXL column (7.8 mm × 300 mm i.d., 13 μm, Tosoh Co., Tokyo, Japan) and a TSK-GRL 3000 PWXL column (7.8 mm × 300 mm i.d., 7 μm, Tosoh Co., Tokyo, Japan) with 0.02 M KH_2_PO_4_ solution as the mobile phase at a flow rate of 0.5 mL/min. The column temperature was kept at 35 °C.

### 3.3. Monosaccharide Composition Analysis

The sample was hydrolyzed (105 °C, 6 h) with 2 mol/L trifluoroacetic acid. After hydrolysis, the residual acid was removed using methanol via repeated vacuum rotary evaporation. Then, a Dionex ICS-3000 ion chromatograph (Dionex Corp. Sunnyvale, CA, USA) was used to analyze the hydrolysate. The chromatographic column was CarboPac^TM^ PA20 column (3 mm × 150 mm). The column temperature was 30 °C. The loading volume was 15 mL. The elution rate was 0.5 mL/min. Gradient elution conditions were as follows: 0–15 min (90% A and 10% B), 15.01–30 min (70% A, 10% B, and 20% C), where A was deionized water, B was 20 mmol/L NaOH, and C was 500 mmol/L NaAc. This method can analyze arabinose, fucose, galactose, galacturonic acid, glucose, glucuronic acid, mannose, and xylose simultaneously.

### 3.4. Fourier Transform Infrared (FT-IR) Spectroscopy Analysis

Freeze-dried polysaccharides were mixed with KBr at a ratio of 1:100, ground, and then pressed into a pellet. FT-IR spectroscopy was recorded (400–4000 cm^−1^) using a Bruker Tensor 27 spectroscopy (Bruker Co. Ltd., Bergisch Gladbach, Germany) with a resolution of 4 cm^−1^ and a scan count of 64 [36].

### 3.5. Morphological Characterization

Scanning electron microscope (SEM) analysis: Take about 10 mg of the freeze-dried sample with a toothpick, place it evenly on the conductive adhesive, and then the sample that was not firmly adhered was blown off and sprayed with gold treatment. The surface morphology was observed under high-vacuum conditions using a Hitachi SU5000 SEM (Hitachi High Technologies, Tokyo, Japan) and the accelerating voltage was 10.0 kV. The image magnification was 2000× and 500× [37].

Atomic force microscopy (AFM) analysis: Cover the 10^−5^ mg/mL sample solution on a freshly cleaved mica sheet. After drying, AFM imaging of the sample was acquired by using a scanning probe microscope in tapping mode (Nanoscope IIIa, Bruker Corporation, Billerica, MA, USA). Nanoscope analysis 1.7 was used to analyze the images (Bruker Corporation, Billerica, MA, USA).

### 3.6. Anti-Inflammatory Activity Evaluation of Polysaccharide Fractions

#### 3.6.1. Cell Culture and Cell Viability Analysis

RAW 264.7 cells were cultured in DMEM containing 10% FBS (37 °C, 5% CO_2_), which was obtained from the BeNa Culture Collection (Beijing, China). After seeding in a 96-well plate (1 × 10^4^ cell/well) and incubating for 24 h, cell viability was detected by using the MTT method (G020-1-2, Nanjing Jiancheng Bioengineering Institute, Nanjing, China).

#### 3.6.2. Measurement of Pro-Inflammatory Mediators

After successive treatment with the sample and lipopolysaccharide (LPS, 1 μg/mL) for 6 h and 24 h, the cell-free culture supernatant of RAW 264.7 cells was collected. The levels of inflammatory mediators were measured according to the manufacturer’s protocol. IL-1β, IL-6, and TNF-α were detected by using ELISA kits (MultiSciences, Hangzhou, Zhejiang, China) [13]. NO was detected by using Griess reagent (Beyotime Institute of Biotechnology, Shanghai, China) [13].

#### 3.6.3. qRT-PCR Analysis

Total RNA was extracted using Trizol reagent (15596026, Invitrogen, Carlsbad, CA, USA), and then the RevertAid First Strand cDNA Synthesis Kit (K1622, Applied Biosystems, Foster City, CA, USA) was used for reverse transcription. The qRT-PCR assay was carried out on a Bio-Rad real-time PCR System (MiniOpticon, Bio-Rad, Hercules, CA, USA) using SYBR^®^ Select Master Mix (4472908, Invitrogen, Carlsbad, CA, USA). The following reaction conditions were performed: Initial hold was 95 °C for 10 min, denaturation was 95 °C for 15 s and run for 40 cycles, and annealing/extension was 60 °C for 1 min. The expression of the gene was calculated using the 2^−ΔΔCt^ method, and the reference gene was *Gapdh*. The primer sequences involved are shown in Supplementary Table S1.

#### 3.6.4. Methylation Analysis

Before the methylation, uronic acids in polysaccharides were reduced following the procedure described [38]. The reduced samples were applied for methylation analysis using the NaOH (sodium hydroxide)-DMSO (dimethylsulphoxide) method. Briefly, the reduced samples after lyophilization were completely dissolved in 500 μL DMSO. Then, 1 mg NaOH and 50 μL CH_3_I (methane iodide) were added successively and kept for 0.5 and 1 h, respectively. The mixture was extracted with methylene chloride and H_2_O three times, then the organic phase was collected and vaporized. The evaporated products were hydrolyzed (121 °C, 1.5 h) by using 100 μL of 2 moL/L trifluoroacetic acid (TFA) and glacial acetic acid was used to terminate the reaction. Finally, the obtained products were washed twice with methanol after drying under nitrogen and then extracted with methylene chloride and H_2_O three times after drying again under nitrogen. The product was analyzed using a GC-MS (mass spectrometry) system (Agilent 7890A/5977B, Agilent Technologies Inc., Santa Clara, CA, USA). Chromatographic parameters: The injection volume was 0.1 μL, the split ratio was 10:1, and the carrier gas was high-purity helium. The heating procedure was as follows: 140 °C for 2.0 min, then 3 °C/min to 230 °C kept at 230 °C for 3 min. MS parameters: The MS system was equipped with an EI and MassHunter workstation and detection was performed in full SCAN mode (30–600 *m*/*z*).

#### 3.6.5. NMR Spectral Analysis

The sample was dissolved in 99.9% D_2_O (deuterium oxide) and then its ^13^C and ^1^H NMR spectra were acquired using a Bruker spectrometer (AVANCE III HD 600, Bruker Corporation, Billerica, MA, USA). The NMR spectra were analyzed using MestReNova 12 software (MestreLab Research, Santiago de Compostela, Spain).

### 3.7. Statistical Analysis

The data were displayed as the means ± standard deviations, which was analyzed using GraphPad Prism 8.0 (GraphPad Software Inc., La Jolla, CA, USA). Significant differences were analyzed using ANOVA with Dunnett’s test (over two groups) and Student’s *t*-test (two groups). The results were considered statistically significant when *p* values were less than 0.05.

## 4. Conclusions

The current study focused on the structural properties and activity of the isolated fractions of polysaccharides from *Sargassum fusiforme* prepared after the UV/H_2_O_2_ treatment. The results indicated that PSF-T2 was separated into two sub-fractions, named PT-0.25 and PT-0.5. Typically, compared with PT-0.25, PT-0.5 not only had higher contents of sulfate radical and fucose, but also had a higher M_W_. In addition, PT-0.25 and PT-0.5 inhibited the secretion of NO, IL-1β, IL-6, and TNF-α and down-regulated the expression of *Tlr4*, *Irak*, *Tnf-α*, *Il-1β*, *Il-6*, and *Il-12*, thus exerting anti-inflammatory activity with a clear dose-dependent effect, among which PT-0.5 displayed better activity. Methylation analysis revealed that PT-0.5 was a complex polysaccharide composed mainly of 4-Fuc*p*, t-Man*p*, 6-Gal*p*, t-Fuc*p*, and 3,4-GlcA*p*. In addition, further study of the relationship between typical structural properties and anti-inflammatory activity needs to be studied, which is important for the preparation of polysaccharides with high anti-inflammatory activity. For structure–activity studies, fucoidan with different sulfate groups, molecular weights, and glycosidic linkage types needed to be prepared.

## Figures and Tables

**Table 1:** Methylation analysis results of PT-0.5.

Residue	Glycosidic Linkage	Methylation	w	Relative Molar Ratio (%)
A	t-Fuc*p*	1,5-di-*O*-acetyl-6-deoxy-2,3,4-tri-*O*-methyl fucitol	293	11.520
B	t-Man*p*	1,5-di-*O*-acetyl-2,3,4,6-tetra-*O*-methyl mannitol	323	13.887
C	t-GalA*p*	1,5-di-*O*-acetyl-2,3,4,6-tetra-*O*-methyl galactitol	323	3.102
D	4-Xyl*p*	1,4,5-tri-*O*-acetyl-2,3-di-*O*-methyl xylitol	307	5.600
E	2-Gal*p*	1,2,5-tri-*O*-acetyl-3,4,6-tri-*O*-methyl galactitol	351	3.333
F	4-Man*p*	1,4,5-tri-*O*-acetyl-2,3,6-tri-*O*-methyl mannitol	351	4.483
G	6-Gal*p*	1,5,6-tri-*O*-acetyl-2,3,4-tri-*O*-methyl galactitol	351	11.942
H	4-Gal*p*	1,4,5-tri-*O*-acetyl-2,3,6-tri-*O*-methyl galactitol	351	3.294
I	4-Fuc*p*	1,4,5-tri-*O*-acetyl-6-deoxy-2,3-di-*O*-methyl fucitol	351	31.852
J	3,4-GlcA*p*	1,3,4,5-tetra-*O*-acetyl-2,6-di-*O*-methyl glucitol	379	10.987

## Data Availability

The data presented in this study are available on request from the corresponding author. The data are not publicly available due to privacy.

## Supplementary Materials

The following supporting information can be downloaded at: https://www.mdpi.com/article/10.3390/md21110561/s1, Table S1: Primer sequences of genes.
