# Supplementary material for: UV/H2O2-Degraded Polysaccharides from Sargassum fusiforme: Purification, Structural Properties, and Anti-Inflammatory Activity
Source: Mar Drugs. 2023 Oct 26;21(11):561. doi: 10.3390/md21110561 (PMC10672335; doi:10.3390/md21110561)
Supplement: Supplementary file 1 [file marinedrugs-21-00561-s001.zip › marinedrugs-2638523-supplementary.pdf]

Table S1 Primer sequences of genes

Gene	Primer (5´-3´)
Trl4	Forward: CAACATCATCCAGGAAGGC
	Reverse:GAAGGCGATACAATTCCACC
Irak	Forward:GTAGCCCAAAGGCATCAGGT
	Reverse:CAAGTTCTTTGGCTGGCCCT
Il-1β	Forward: GAAATGCCACCTTTTGACAGTG
	Reverse: TGGATGCTCTCATCAGGACAG
Il-6	Forward: CTGCAAGAGACTTCCATCCAG
	Reverse: AGTGGTATAGACAGGTCTGTTGG
Il-12	Forward: CTGGAGCACTCCCCATTCCTA
	Reverse: GCAGACATTCCCGCCTTTG
Tnf-α	Forward: CAGGCGGTGCCTATGTCTC
	Reverse: CGATCACCCCGAAGTTCAGTAG
Gapdh	Forward: CTCGTCCCGTAGACAAAATGGT
	Reverse: GAGGTCAATGAAGGGGTCGTT
